# Sperm cryopreservation of *Prochilodus brevis* using different concentrations of non-permeable cryoprotectants

**DOI:** 10.1590/1984-3143-AR2021-0083

**Published:** 2022-02-04

**Authors:** Thais Maia Torres, Priscila Silva de Almeida-Monteiro, Renata Vieira do Nascimento, Vanessa Alves Pereira, Yasmim Maia Ferreira, Jessica Sales Lobato, Romulo Roberto Ribeiro Pinheiro, Yara Silvino Sales, Assis Rubens Montenegro, Carminda Sandra Brito Salmito-Vanderley

**Affiliations:** 1 Programa de Pós-graduação em Ciências Veterinárias, Laboratório de Biotecnologia da Reprodução de Peixes, Universidade Estadual do Ceará, Fortaleza, CE, Brasil; 2 Laboratório de Biotecnologia da Reprodução de Peixes, Universidade Estadual do Ceará, Fortaleza, CE, Brasil

**Keywords:** cryopreservation, extender, fish, semen

## Abstract

The action of substances with non-permeable cryoprotectant potential, besides glucose, has not yet been studied for the species *Prochilodus brevis*. The objective of this work was to evaluate the action of four non-permeable cryoprotectants on this species sperm cryopreservation. Five pools were cryopreserved in a solution of 5% glucose and 10% dimethyl sulfoxide (Me_2_SO) associated or not (control) with cryoprotectants egg yolk (5, 10 or 12%), soy lecithin (2.5, 7.5 or 10%), sucrose (5, 10 or 20%) and lactose (5, 8 or 15%). After thawing, samples were evaluated for sperm kinetics (total motility, motility duration, velocities, and wobble - WOB), morphology and membrane and DNA integrity. The treatments containing egg yolk improved significantly (P<0.05) results when compared the control for the membrane integrity parameter. When compared to other treatments, egg yolk, at any concentration, presented higher results (P<0.05) for membrane integrity, total motility, curvilinear velocity (VCL) and average path velocity (VAP) parameters. Egg yolk also showed the best results for WOB, but it did not differ from 5% and 8% lactose and 5% and 20% sucrose. Soy lecithin had the lowest percentages of morphologically normal sperm (P<0.05), while the other treatments did not differ from each other. There was no difference regarding DNA integrity data. Thus, 5% egg yolk is indicated as a non-permeable cryoprotectant for *P. brevis*, in association with 5% glucose and 10% Me_2_SO.

## Introduction

*Prochilodus brevis* is a teleost fish native to the Brazilian semiarid region, which occupies the entire Northeast region of Brazil (Chellappa et al., 2009). Due to its detritivorous habit, this species is of a great ecological importance, as it drives biomass from lower to higher levels of the food chain (Mcintyre et al., 2007; Gurgel et al., 2012). During the rainy season *P. brevis* migrate towards the river headwaters to reproduce, a process known as piracema (Araújo, 1998; Gurgel et al., 2012). However, the construction of dams and the rain scarcity hazard this phenomenon, hindering its reproduction and threatening its preservation in nature (Nascimento et al., 2010). Moreover, *P. brevis* roe, known as “caviar do sertão” (backwoods caviar), is a highly appreciated delicacy, which gives the species an economic importance either. However, this leads the capture of females during their reproductive period, which makes their reproduction in natural environment even more difficult. Therefore, *P. brevis* captive breeding is an excellent alternative as it enhances the roe commercialization, in addition to minimizing the wild female capture while in its reproductive age. Hence, the development of reproductive biotechnologies, such as sperm cryopreservation, is relevant to facilitate the species artificial reproduction, as the sperm can be kept frozen for an indeterminate time, which may avoid possible issues related to reproductive asynchrony between males and females, in addition to reduce the number of male broodstock in fish farming stations (Viveiros and Godinho, 2009).

Sperm cryopreservation has been increasingly studied for application in fish reproduction, and which can be used for commercial or conservation purposes (Shimoda, 2004; Magnotti et al., 2016). However, during cell freezing, water inside the system is changing from liquid to solid state, which may cause damage to the cell if the procedure is not controlled and well observed (Mazur, 1984). To carry out this technique, it is necessary to use a cryosolution, composed of cryoprotectants, to minimize cell damage (Kopeika et al., 2007). The cryoprotectants can act internally (permeable) or externally (non-permeable) to the cell, and both types may be applied in fish sperm cryopreservation (Kopeika et al., 2007). This combination of cryoprotectants may bring benefits to post-thawed sperm quality as it reduces the damage caused by intra and extracellular ice crystals (Mazur, 1984; Fowler and Toner, 2006). Regarding *P. brevis* species, 5% glucose, as non-permeable, and 10% dimethyl sulfoxide (Me_2_SO), as permeable, compose the ideal solution to cryopreserve its sperm (Nunes et al., 2016, 2019; Almeida-Monteiro et al., 2017).

Concerning the study of non-permeable cryoprotectants, they are usually substances with high molecular weight or rich in lipoproteins (Aires et al., 2003). Moreover, they can act by covering the cell surface and stabilizing the sperm membrane, as well as dehydrating the cell or replacing phospholipids lost to cold shock (Watson, 2000; Bucak et al., 2013). Thus, among non-permeable substances already tested for fish sperm cryopreservation, besides glucose, the following stand out: egg yolk (Carolsfeld et al., 2003; Muchlisin et al., 2020); soy lecithin (Yildiz et al., 2013; Lopes, 2019); and disaccharides, such as lactose (Golshahi et al., 2018; Rusco et al., 2019) and sucrose (Felizardo et al., 2016, 2010; Golshahi et al., 2018). As stated, egg yolk is one of the most used non-permeable cryoprotectant. It brings satisfactory results for fish sperm cryopreservation, as it protects the membrane integrity against damage caused by the cold (Felizardo et al., 2016), since it acts as a shield against cold shock and extender toxicity (Muchlisin et al., 2020). However, some studies have been developed to replace the use of animal source cryoprotectants. Thus, low density lipoproteins (LDL) of vegetal source, such as soy lecithin, are an option. In sperm cells, lecithin acts as an exogenous source of phospholipids, which can interact with the plasma membrane, preventing its lysis and repairing damage caused by the freezing process (Aires et al., 2003). Besides these lipoproteins, carbohydrates such as lactose and sucrose might also be used as non-permeable cryoprotective agents, since sugars act by stabilizing the sperm membrane through an interaction with the polar heads of its phospholipids, as well as by decreasing the lipid phase change temperature and by providing energy to the sperm during incubation (Aisen et al., 2002).

However, the action of non-permeable cryoprotectants associated with the usually applied solution - Glucose and Me_2_So - has not yet been studied for *P. brevis* sperm. Therefore, the aim of this study was to evaluate the effect of egg yolk, soy lecithin, sucrose, and lactose on this species post-thawed sperm parameters such as kinetics (total motility, motility duration, velocities, and wobble), morphology and membrane and DNA integrity.

## Methods

### Experimental design

The experiment was carried out at the Fish Reproduction Biotechnology Laboratory (LBRP), located on the Itaperi campus of the State University of Ceará (3°47'36.2”S; 38°33'30.1”W). In this study, we evaluated the action of four non-permeable cryoprotectants, at three different concentrations, on *P. brevis* sperm cryopreservation. The cryoprotectants and their tested concentrations were egg yolk (5, 10 or 12%); soy lecithin (2.5; 7.5 or 10%); sucrose (5, 10 or 20%) and lactose (5, 8 or 15%).

### Fish and sperm collection

Nineteen *P. brevis* males were used. They were belonging to the LBRP herd, kept in 7100-liter fiberglass tanks, and fed daily with commercial food. Those with characteristics indicative of reproductive maturity, such as hyperemic urogenital papilla and easy semen release, were selected for the experiment.

The selected males were first induced to sperm with two doses of carp pituitary extract (EHC), with an interval of eight hours between them. The first dose consisted of 0.4 mg EHC/kg and the second 4.0 mg EHC/kg. The sperm collection was carried out 15 hours after the second dose.

Males were sedated with a eugenol solution (Sigma-Aldrich®), at a ratio of 1:10:10000 (eugenol: absolute alcohol: tank water) to reduce stress. Each fish was submerged in this solution until unsteadiness. After that, the semen was collected through abdominal massage in 1 mL sterile syringes. Contamination by water, feces or urine was avoided. The collected semen was transferred to graduated polyethylene tubes and kept in ice until processing.

Samples were evaluated for sperm motility, and those with rates below 90% were not used. Five pools (replicates; *n* = 5) were formed (4 independent males per pool) and directed to analysis and subsequent freezing.

### Sperm analysis

To assess the sperm concentration, aliquots of *in natura* semen from each pool (*n* = 5) were fixed by a 4% formaldehyde citrate solution, in a 1:4000 proportion (1 µL semen:4 mL fixative). From this mixture, 20 µL were placed in a Neubauer chamber which five quadrants were analyzed under an optical microscope at 400x magnification by sperm cells counting.

An aliquot from each pool (*n* = 5) was analyzed for sperm kinetics. For this purpose, 1 µL of semen was mixed with 100 µL of activating solution (NaCl 125 mOsm) in the Makler chamber (analyzed approximately 15 seconds after activation). Analyzes were performed using the Computer-Assisted Sperm Analysis (CASA), with Sperm Class Analyzer software (SCA, Microptics – Barcelona - Spain, version 3.2) using the settings indicated for fish. The kinetic parameters evaluated were total motility (%); curvilinear velocity (VCL - μm/s); straight line velocity (VSL - μm/s); average path velocity (VAP - μm/s) and Wobble (WOB - %).

For sperm morphology, an aliquot from each pool (*n* = 5) was fixed in a 4% formaldehyde citrate solution, at a 1:10 (semen:fixer) ratio. Then, it was dyed with Rose Bengal staining solution (20 mL distilled water, 0.58 g sodium citrate tribasic, 0.8 mL formaldehyde, 0.3 g rose bengal), at a ratio of 3:20 (staining solution:fixed sperm). This mixture was placed on a histological slide for the smearing. Finally, samples were analyzed under an optical microscope at 400x magnification. One hundred sperm cells per slide were classified as normal or damaged according to Miliorini et al. (2011), and two slides per sample were evaluated.

For sperm membrane integrity evaluation, the eosin-nigrosin staining method was applied (Blom, 1950). A mixture and smear of 5 µL of sperm with 10 µL of eosin and 10 µL of nigrosin was performed (1:2:2 ratio - semen:eosin:nigrosin). One slide per pool (*n* = 5) was evaluated under a light microscope (400x) by sliding through visual fields until reaching the count of 200 sperm cells, where colorless sperm were considered to have an intact membrane, while those stained in pink or red had a ruptured membrane.

The sperm DNA integrity was assessed using the SCD (Sperm Chromatin Dispersion) test, which analyzes the rate of sperm chromatin fragmentation, following the methodology of Fernandez et al. (2005) adapted and described by Almeida-Monteiro et al. (2020). Two hundred and fifty sperm cells per pool (*n* = 5) were analyzed by sliding through visual fields until reaching this count, and classified as having intact DNA (when a halo was observed around sperm head) and fragmented DNA (when no halo was observed).

### Sperm freezing

An aliquot of each five pools (replicates; *n* = 5) was diluted (1:9 - semen:extender) in 5% Glucose and 10% Me_2_SO (standard cryosolution for *P.brevis*) associated or not (control) with four non-permeable cryoprotectants in three different concentrations each: Egg yolk (YOLK – 5, 10 or 12%) Soy lecithin (LEC – 2.5, 7.5 or 10%), Lactose (LAC – 5, 8 or 15%) or Sucrose (SUC – 5, 10 or 20%), what equals 12 treatments and the control group.

After dilution, the samples were cryopreserved. For this matter, each treatment was filled in two 0.25 mL French straws, which were sealed with polyvinyl alcohol and kept under refrigeration (10 ºC) for 10 minutes. Then, the straws were frozen in a dry shipper (-176 °C) for 15 minutes and stored in liquid nitrogen (-196 °C).

The samples were thawed six months later, in a water bath at 30°C for 16 seconds (Nunes et al., 2016), and analyzed for the same parameters described above. In addition, they were also analyzed for motility duration (*n* = 5). For this evaluation, 1 µL of semen was placed on the Makler chamber and activated with 100 µL of 125 mOsm NaCl. A stopwatch was started at the time of sperm activation until all movement in the analyzed visual field had ceased (one visual field per replicate for each treatment).

### Statistical analysis

The data were submitted to the Shapiro-Wilk and Bartlett tests to verify the normal distribution of residues and homoscedasticity, respectively. Then, data underwent a logarithmic transformation to fit the ANOVA, which was performed using SAS software (PROC GLM; 2002). Comparisons between the means of treatments were made using Student Newman Keuls test (SNK), while between treatments and control were performed using the Dunnet test. All data were expressed as mean ± standard deviation of the means, considering a significance level of at least 5% (P < 0.05).

Regarding the membrane integrity parameter, the data were submitted to ANOVA (P <0.05) using the MASS package (Venables and Ripley, 2002) in R (R Core Team, 2020). When significant, Dunnett post-hoc (P <0.05) was used to compare mean treatment levels versus respective controls using the DescTools package (Signorell, 2021). To verify significant mean difference between treatments, data were submitted to ANOVA and Tukey's post-hoc (P <0.05) was used to pairwise comparison using the agricolae package (Mendiburu and Yaseen, 2020).

### Animal care

The study was approved by the Ethics Committee for Animal Use of the State University of Ceará (Protocol number: 340620/2020).

## Results

### Fresh sperm

Pools formed with *in natura* sperm had an average concentration of 23.96 × 10^9^ sperm/mL. Regarding total motility rate, an average of 97.28 ± 1.50% was observed. The analyzed velocities, VCL, VSL and VAP, had averages of 90.96 ± 8.92 µm/s, 45.54 ± 3.50 µm/s and 67.64 ± 3.19 µm/s, respectively. Fresh sperm presented an average WOB of 74.80 ± 6.06%. A percentage of 93.40 ± 1.34% of morphologically normal sperm was observed, 96.70 ± 0.45% of sperm with intact membrane and 95.48 ± 2.23% with DNA integrity.

### Post-thawed sperm

Concerning total motility rate of post-thawed sperm, treatments containing 5% and 12% egg yolk did not show significant difference compared to the control group, while all other treatments presented lower motility rate than control (P<0.05; Table 1). Furthermore, when the treatments were compared to each other, those containing egg yolk (5%, 10% and 12%) did not differ from each other and had higher motility than all others (P<0.05; Table 2). Treatments containing 5%, 8% and 15% lactose did not differ from each other, and their two lowest concentrations were significantly higher than treatments containing soy lecithin (any concentration) or 10% and 20% sucrose (P<0.05; Table 2). In turn, 15% lactose and 5% sucrose were similar both to treatments containing soy lecithin and 10% and 20% sucrose, and to other concentrations of lactose (Table 2).

**Table 1:** Sperm kinetic parameters [motility (%), motility duration (s), curvilinear velocity (VCL; µm/s), straight line velocity (VSL; µm/s) and wobble (WOB; %)] of *P .brevis* sperm (*n* = 5, pool replicates) cryopreserved with a solution composed of 5% glucose and 10% dimethyl sulfoxide (Me_2_SO) associated with non-permeable cryoprotectants egg yolk (YOLK - 5, 10 and 12%), soy lecithin (LEC - 2.5, 7.5 and 10%), lactose (LAC - 5, 8 and 15%) or sucrose (SUC - 5, 10 and 20%) and its comparison with the control group (5% glucose with 10% Me_2_SO).

	**Motility (%)**	**Motility duration (s)**	**VCL (µm/s)**	**VSL (µm/s)**	**WOB (%)**
Control	62.30 ± 13.14	52.42 ± 34.44	38.96 ± 12.98	17.60 ± 9.33	62.02 ± 12.87
YOLK 5%	49.00 ± 7.96	39.61 ± 7.27	35.68 ± 1.72	14.14 ± 1.78	58.60 ± 3.46
YOLK 10%	47.08 ± 8.72*	42.55 ± 16.12	32.34 ± 3.98	13.06 ± 3.53	60.58 ± 5.52
YOLK 12%	51.68 ± 7.48	49.60 ± 21.76	34.10 ± 6.70	13.26 ± 4.06	59.56 ± 4.65
LEC 2.5%	20.64 ± 2.13***	09.79 ± 13.99	17.88 ± 1.17***	2.68 ± 0.64***	35.40 ± 2.69***
LEC 7.5%	22.84 ± 4.40***	14.66 ± 28.87*	17.78 ± 1.62***	2.74 ± 0.58***	36.82 ± 1.94***
LEC 10%	21.84 ± 2.86***	01.10 ± 0.22**	17.66 ± 1.39***	2.86 ± 0.77***	36.24 ± 4.90***
LAC 5%	36.38 ± 12.86***	40.72 ± 40.72	25.08 ± 7.76*	7.62 ± 5.42	47.26 ± 13.40
LAC 8%	36.30 ± 10.82***	37.81 ± 30.58	23.56 ± 6.15**	7.68 ± 5.36	49.14 ± 12.58
LAC 15%	25.50 ± 2.07***	29.26 ± 25.16	18.64 ± 0.54***	3.46 ± 0.91***	37.82 ± 3.46**
SUC 5%	27.62 ± 4.96***	37.40 ± 45.43	22.22 ± 4.37***	5.98 ± 3.44*	44.54 ± 11.44*
SUC 10%	24.40 ± 3.90***	25.11 ± 35.08	19.22 ± 3.63***	4.10 ± 3.09***	40.34 ± 10.26**
SUC 20%	23.20 ± 1.78***	20.17 ± 37.00	19.03 ± 1.38***	03.02 ± 0.30	44.75 ± 5.93

* Indicates statistical difference between the control (without non-permeable cryoprotectant) and the treatment. P< 0.05. Mean ± SD;

** Indicates statistical difference between the control (without non-permeable cryoprotectant) and the treatment. P< 0.01. Mean ± SD;

*** Indicates statistical difference between the control (without non-permeable cryoprotectant) and the treatment. P< 0.001. Mean ± SD.

**Table 2:** Sperm kinetic parameters [motility (%), motility duration (s), curvilinear velocity (VCL; µm/s), straight line velocity (VSL; µm/s), average path velocity (VAP; µm/s) and wobble (WOB; %)] of *P. brevis* sperm (*n* = 5, pool replicates) cryopreserved with a solution composed of 5% glucose and 10% dimethyl sulfoxide (Me_2_SO) associated with non-permeable cryoprotectants egg yolk (YOLK - 5, 10 and 12%), soy lecithin (LEC - 2.5, 7.5 and 10%), lactose (LAC - 5, 8 and 15%) or sucrose (SUC - 5, 10 and 20%).

	**Motility (%)**	**Motility duration (s)**	**VCL (µm/s)**	**VSL (µm/s)**	**VAP (µm/s)**	**WOB (%)**
YOLK 5%	49.00 ± 7.96^a^	39.61 ± 7.27^a^	35.68 ± 1.72^a^	14.14 ± 1.78^ab^	20.92 ± 2.05^a^	58.60 ± 3.46^a^
YOLK 10%	47.08 ± 8.72^a^	42.55 ± 16.12^a^	32.34 ± 3.98^a^	13.06 ± 3.53^abc^	19.76 ± 4.02^a^	60.58 ± 5.52^a^
YOLK 12%	51.68 ± 7.48^a^	49.60 ± 21.76^a^	34.10 ± 6.70^a^	13.26 ± 4.06^abc^	20.54 ± 5.21^a^	59.56 ± 4.65^a^
LEC 2.5%	20.64 ± 2.13^c^	09.79 ± 13.99^ab^	17.88 ± 1.17^b^	2.68 ± 0.64^d^	06.34 ± 0.84^b^	35.40 ± 2.69 ^b^
LEC 7.5%	22.84 ± 4.40^c^	14.66 ± 28.87^ab^	17.78 ± 1.62 ^b^	2.74 ± 0.58^d^	06.54 ± 0.89^b^	36.82 ± 1.94^b^
LEC 10%	21.84 ± 2.86^c^	01.10 ± 0.22^b^	17.66 ± 1.39^b^	2.86 ± 0.77^d^	06.42 ± 1.19^b^	36.24 ± 4.90^b^
LAC 5%	36.38 ± 12.86^b^	40.72 ± 40.72^a^	25.08 ± 7.76 ^b^	7.62 ± 5.42^cd^	12.64 ± 6.99^b^	47.26 ± 13.40^ab^
LAC 8%	36.30 ± 10.82^b^	37.81 ± 30.58^a^	23.56 ± 6.15^b^	7.68 ± 5.36^bcd^	12.20 ± 6.25^b^	49.14 ± 12.58^ab^
LAC 15%	25.50 ± 2.07^bc^	29.26 ± 25.16^a^	18.64 ± 0.54 ^b^	3.46 ± 0.91^d^	07.04 ± 0.72^b^	37.82 ± 3.46^b^
SUC 5%	27.62 ± 4.96 ^bc^	37.40 ± 45.43^a^	22.22 ± 4.37^b^	5.98 ± 3.44^d^	10.28 ± 4.26^b^	44.54 ± 11.44^ab^
SUC 10%	24.40 ± 3.90^c^	25.11 ± 35.08^ab^	19.22 ± 3.63^b^	4.10 ± 3.09^d^	8.04 ± 3.70^b^	40.34 ± 10.26^b^
SUC 20%	23.20 ± 1.78^c^	20.17 ± 37.00^ab^	19.03 ± 1.38 ^b^	03.02 ± 0.30^d^	07.03 ± 0.17^b^	44.75 ± 5.93^ab^

Different letters indicate statistical difference between treatments. P<0.05. Mean ± SD.

Regarding motility duration, all treatments were similar to the control (P>0.05), except 7.5% and 10% lecithin, which presented lower results (P<0.05; Table 1). When the treatments were compared to each other, those containing egg yolk and lactose, at any concentration, and 5% sucrose showed the best results (P<0.05) and did not differ from each other (Table 2). The lowest motility duration results were obtained when 10% soy lecithin was used. The other treatments did not show significant differences (Table 2).

For the VCL parameter, the treatments containing egg yolk, at any concentration, were similar to the control, while all other treatments presented lower VCL than this group (P<0.05; Table 1). Moreover, egg yolk, at any concentration, was responsible for promoting the highest VCL and VAP values when compared to any of the other non-permeable cryoprotectants (P<0.05). The other treatments did not differ from each other (Table 2).

For the VSL parameter, the treatments containing egg yolk, at any concentration, and 5% and 8% lactose did not differ from the control (Table 1). The other treatments were significantly lower than the control group (P<0.05; Table 1). The three egg yolk concentrations did not differ from each other (Table 2) and showed the best results. Furthermore, the treatments containing 5% and 8% lactose were similar both to the highest egg yolk concentrations (10% and 12%) and to the other treatments (Table 2). Sucrose and lecithin, at any concentration, and 15% lactose did not differ from each other (Table 2).

Regarding WOB, the control group did not differ from treatments containing egg yolk, at any concentration, nor from 5% and 8% lactose and 20% sucrose (Table 1). When the treatments were compared with each other, egg yolk, at all concentrations, showed the best results (P<0.05; Table 2). The lowest percentages were obtained with soy lecithin at any concentration, 15% lactose and 10% sucrose (P<0.05). The remaining treatments did not differ from those that presented neither the highest nor the lowest WOB results (Table 2).

As for morphological analysis, only tail related defects were detected: coiled, bent, and fractured tail. Of these, the bent tail damage was the most found. Morphological analysis did not result statistical difference between the control group and any treatment (Table 3). However, when the treatments were compared to each other, egg yolk, lactose, and sucrose, at any concentration, resulted in the highest percentages of normal sperm (P<0.05) and did not differ from each other (Table 4). Thus, soy lecithin showed the lowest results for this parameter, with no difference between its concentrations. Concerning DNA integrity, no statistical difference was observed either between treatments and the control group, or between treatments (Table 3 and 4).

**Table 3:** DNA Integrity (%), Normal Morphology (%) and Membrane Integrity (%) of P.brevis sperm (n = 5, pool replicates) cryopreserved with a solution composed of 5% glucose and 10% dimethyl sulfoxide (Me2SO) associated with non-permeable cryoprotectants egg yolk (YOLK - 5, 10 and 12%), soy lecithin (LEC -2.5, 7.5 and 10%), lactose (LAC - 5, 8 and 15%) or sucrose (SUC - 5, 10 and 20%) and their comparison with the control group (5% glucose with 10% Me2SO).

	**DNA integrity (%)**	**Normal morphology (%)**	**Membrane integrity (%)**
Control	86.44 ± 2.25	87.20 ± 2.02	64.80 ± 5.86
YOLK 5%	89.76 ± 5.94	81.20 ± 4.56	91.20 ± 3.03***
YOLK 10%	86.64 ± 6.53	82.60 ± 2.01	89.60 ± 2.30***
YOLK 12%	88.58 ± 5.48	81.20 ± 3.73	84.80 ± 12.31**
LEC 2.5%	86.88 ± 5.78	60.60 ± 6.20	01.80 ± 0.57***
LEC 7.5%	89.04 ± 3.13	57.70 ± 9.61	01.90 ± 1.74***
LEC 10%	86.48 ± 4.39	59.70 ± 5.96	03.60 ± 1.14***
LAC 5%	86.64 ± 4.11	83.30 ± 2.66	53.30 ± 4.63**
LAC 8%	85.92 ± 7.70	80.00 ± 8.83	51.20 ± 7.15**
LAC 15%	92.28 ± 4.68	80.10 ± 1.94	52.00 ± 1.22**
SUC 5%	87.84 ± 3.56	84.80 ± 2.54	50.90 ± 23.16
SUC 10%	87.20 ± 6.16	81.90 ± 2.24	39.60 ± 5.86*
SUC 20%	87.10 ± 3.21	83.25 ± 1.85	21.50 ± 4.12***

* Indicates statistical difference between the control (without non-permeable cryoprotectant) and the treatment. P< 0.05. Mean ± SD;

** Indicates statistical difference between the control (without non-permeable cryoprotectant) and the treatment. P< 0.01. Mean ± SD;

*** Indicates statistical difference between the control (without non-permeable cryoprotectant) and the treatment. P< 0.001. Mean ± SD.

**Table 4:** DNA Integrity (%), Normal Morphology (%) and Membrane Integrity (%) of *P.brevis* sperm (*n* = 5, pool replicates) cryopreserved with a solution composed of 5% glucose and 10% dimethyl sulfoxide (Me_2_SO) associated with non-permeable cryoprotectants egg yolk (YOLK - 5, 10 and 12%), soy lecithin (LEC -2.5, 7.5 and 10%), lactose (LAC - 5, 8 and 15%) or sucrose (SUC - 5, 10 and 20%).

	**DNA integrity (%)**	**Normal morphology (%)**	**Membrane integrity (%)**
YOLK 5%	89.76 ± 5.94	81.20 ± 4.56^a^	91.20 ± 3.03ª
YOLK 10%	86.64 ± 6.53	82.60 ± 2.01^a^	89.60 ± 2.30ª
YOLK 12%	88.58 ± 5.48	81.20 ± 3.73^a^	84.80 ± 12.31ª
LEC 2.5%	86.88 ± 5.78	60.60 ± 6.20^b^	01.80 ± 0.57^e^
LEC 7.5%	89.04 ± 3.13	57.70 ± 9.61^b^	01.90 ± 1.74^e^
LEC 10%	86.48 ± 4.39	59.70 ± 5.96^b^	03.60 ± 1.14^e^
LAC 5%	86.64 ± 4.11	83.30 ± 2.66^a^	53.30 ± 4.63^bc^
LAC 8%	85.92 ± 7.70	80.00 ± 8.83^a^	51.20 ± 7.15^bc^
LAC 15%	92.28 ± 4.68	80.10 ± 1.94^a^	52.00 ± 1.22^bc^
SUC 5%	87.84 ± 3.56	84.80 ± 2.54^a^	50.90 ± 23.16^bc^
SUC 10%	87.20 ± 6.16	81.90 ± 2.24^a^	39.60 ± 5.86^cd^
SUC 20%	87.10 ± 3.21	83.25 ± 1.85^a^	21.50 ± 4.12^d^

Different letters indicate statistical difference between treatments. P<0.05. Mean ± SD. Absence of letters indicate no statistical difference between treatments. P>0.05. Mean ± SD.

As for membrane integrity, the treatments containing 5%, 10% and 12% egg yolk provided more protection to plasma membrane than the control group (P<0.001; P<0.001; P<0.01, respectively; Table 3). The other treatments, containing any of the non-permeable cryoprotectants at any concentration, provided less protection than the control (P<0.01), except for 5% sucrose, which was statistically similar (Table 3). When the treatments were compared with each other, the egg yolk at any concentration provided better results than the others (P<0.05; Table 4). Soy lecithin, at any concentration, was responsible for the lowest values. While lactose did not differ from each other or to 5% and 10% sucrose (Table 4).

**Figure 1:**
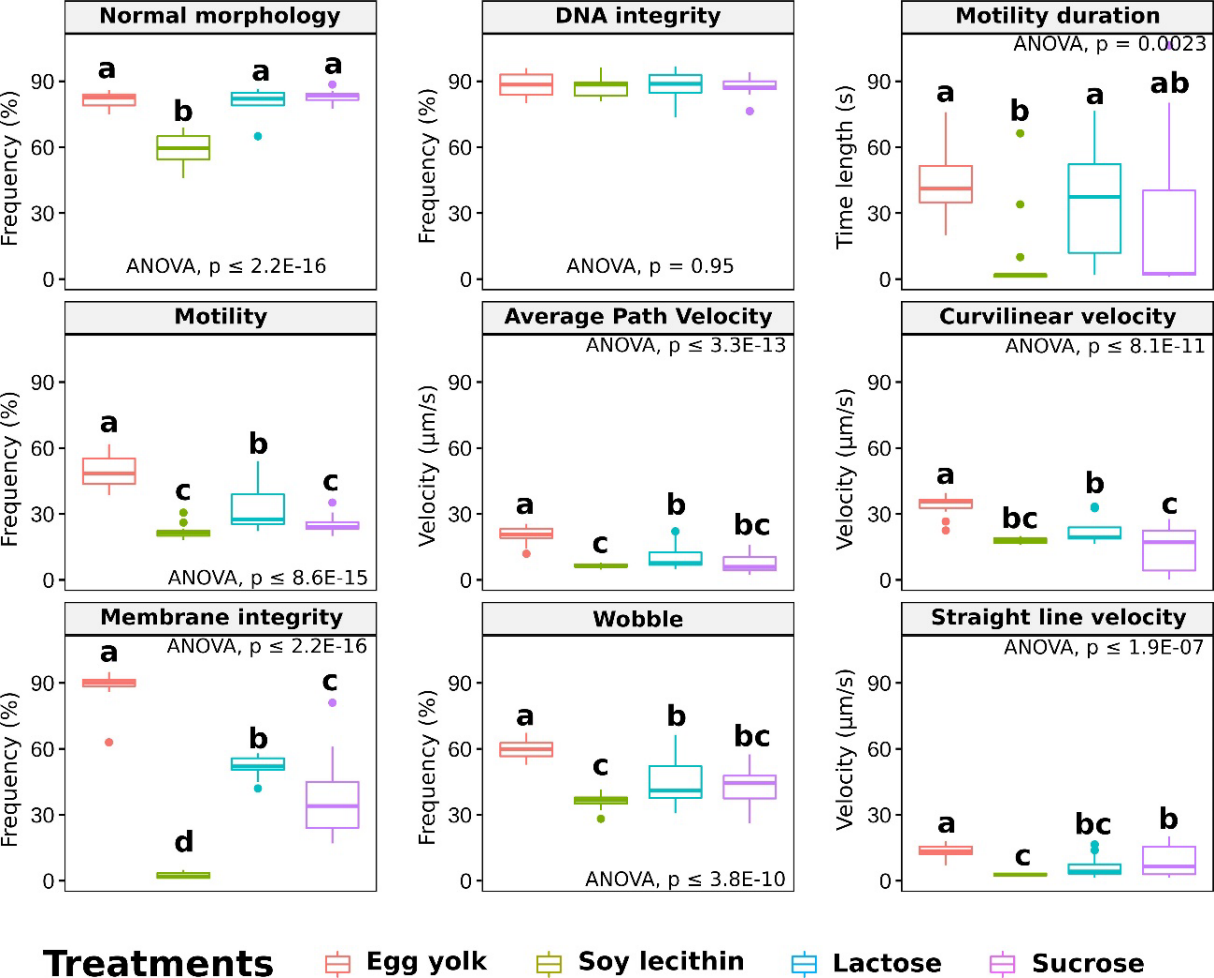
Sperm kinetics parameters [motility (%), motility duration (s), curvilinear velocity (VCL; µm/s), straight-line velocity (VSL; µm/s), average path velocity (VAP; µm/s) and wobble (WOB; %)], DNA integrity (%), Normal morphology (%) and Membrane integrity (%) of *P. brevis* sperm (*n* = 5, pool replicates) cryopreserved with 5% glucose and 10% dimethyl sulfoxide (Me_2_SO) associated with non-permeable cryoprotectants egg yolk, soy lecithin, lactose or sucrose, regardless of the concentration used (ANOVA *post-hoc* Tukey, p ≤ 0,05).

When the treatments were analyzed regardless of the concentration used, the egg yolk showed higher results to all the other treatments for total motility, VCL, VSL, VAP, WOB and membrane integrity (P<0.05; Figure 1). For morphology and motility duration, egg yolk, lactose and sucrose were similar (P>0.05; Figure 1) and soy lecithin showed the lowest results, not only differing from sucrose for motility duration. Regarding DNA integrity, there was no difference between the non-permeable cryoprotectants used (P>0.05; Figure 1).

## Discussion

To achieve a successful cryopreservation, this biotechnology must be studied in a species-specific manner (Kopeika et al., 2007). However, several fish species, such as *P. brevis* still lack studies for its breeding. Thus, finding the best non-permeable cryoprotectant for this species cryopreservation is an important step. This study showed that the use of substances with this function can generate promising results.

Sperm motility is one of the most important reproductive parameters to be evaluated for fish reproduction, as it has a high correlation with parameters that indicate reproductive success (Cosson, 2019; Liu et al., 2007; Dziewulska et al., 2011; Sanches et al., 2015). In a study with *Prochilodus lineatus*, egg yolk, in association with methanol, presented the highest motility rates, while lactose improved relevant sperm parameters when Me_2_SO was used (Felizardo et al., 2010). In the present study, the treatments containing egg yolk (in association with Me_2_SO) presented the best motility rates compared to other non-permeable cryoprotectants. This result illustrates the species-specific characteristic that these substances have, since species of the same genus can present divergent results (Kopeika et al., 2007).

Egg yolk is part of the sperm cryopreservation protocol of fish species such as *Colossoma macropomum* (Maria et al., 2011; Carneiro et al., 2012) and *Prochilodus magdalenae* (Martínez and Pardo, 2013; Garcia et al., 2015). The positive results obtained when using this substance can be attributed to two main factors, which act at different times and with different goals: The resistance factor, which protects the cell against cold shock during freezing; and the storage factor, which acts to maintain the sperm motility, membrane integrity and fertilizing capacity during the storage period (Kampschmidt et al., 1953; Yildiz et al., 2013).

In the present research, treatments containing soy lecithin did not show favorable results for total motility. High concentrations of soy lecithin were considered toxic and led to a decrease in motility rate, motility duration, membrane integrity and fertilization rate of *Cyprinus carpio* post-thawed sperm (Yildiz et al., 2013). This result was attributed by the authors to this substance high viscosity at concentrations higher than 15%. However, in the same study, when a 10% concentration was used, lecithin showed satisfactory results, similar to those of egg yolk. In a study testing soy lecithin in *C. macropomum* sperm cryopreservation, the concentrations 2.5%, 5% and 7.5% had similar kinetic results to those of egg yolk, while 10% lecithin increased viscosity and led to losses in sperm motility (Lopes, 2019). Although lower concentrations of lecithin were used in our research, it was still observed high viscosity of the freezing medium when adding it, which may have led to losses in motility.

The treatments containing egg yolk and lactose, at any concentration, and sucrose at the lowest concentration, showed the best results for motility duration in the present study. Sugars are good cryoprotective agents because they act in different ways: interacting with the polar heads of the plasma membrane phospholipids, which leads to membrane stabilization; decreasing the lipid phase change temperature; and providing energy to sperm cells (Aisen et al., 2002). This last characteristic may be the main responsible for promoting the longest periods of motility found in this study. Felizardo et al. (2016) observed that lactose was the non-permeable cryoprotectant with the longest sperm motility durations in *Brycon orbignyanus*. Furthermore, when non-permeable cryoprotectants were tested on *Salmo cettii* sperm freezing, 10% egg yolk and 0.1 M sucrose presented the best results for this parameter (Rusco et al., 2019). In our work, sucrose at higher concentrations formed a precipitate, which may have caused decrease in sperm kinetics.

VCL is a kinetic parameter positively correlated with fertilization rate in fish. It is necessary that the sperm perform circular movements around the oocyte searching for its entry opening, the micropyle (Sanches et al., 2015). In addition to the VCL, a positive correlation was found between the VSL and the fertilization rate for *Salmo salar* (Figueroa et al., 2016). For the same species, a correlation was observed between VAP (as well as VCL) and fertilization success, which was measured by the number of embryos with optic vesicle (Dziewulska et al., 2011). Therefore, substances that improve or maintain sperm velocities (VCL, VSL and VAP) must be wanted, and that happened when using egg yolk as a non-permeable cryoprotectant.

Among sperm analysis parameters, WOB is little discussed, and its influence on sperm quality, as well as its correlation with fertilization rate, is still poorly understood. This analysis indicates the efficiency of sperm forward displacement (Ingermann et al., 2011), therefore, higher rates are preferred. In this study, the treatments containing egg yolk also stood out for this parameter, presenting the highest rates.

Sperm conservation procedures can lead to sperm morphological damage (Fauvel et al., 2010), and the substances used may be responsible for that. Soy lecithin, at any concentration, presented the lowest rates of sperm with normal morphology, between 57% and 60%. This may indicate that it not only failed to protect cells against damage caused by cold shock but could also have been toxic to *P. brevis* sperm, increasing the injuries. Furthermore, around 50% of sperm anomalies is considered critical, as it can influence the fertilization rate (Miliorini et al., 2011). In this study, the anomalies observed were mostly from tail, such as bent, curled, corrugated and loose tail. Morphological changes can cause the sperm to acquire an oscillatory movement, which hinders its movement and brings motility loss (Kavamoto et al., 1999).

In the present work, DNA integrity was analyzed using the test that verifies sperm chromatin dispersion. This is an important analysis since, after cryopreservation, despite being mobile and morphologically normal, sperm may have their DNA fragmented (Almeida-Monteiro et al., 2020). This means that, even if they are able to fertilize the oocyte, they can later lead to dysfunctions in the development of embryos and larvae (Figueroa et al., 2016). However, in this study, there was no difference for this parameter between the cryoprotectants tested. This indicates that all the substances were able to protect the sperm DNA.

Regarding membrane integrity, previous studies with *P. brevis* sperm cryopreservation resulted percentages of 56.15 ± 3.46%; 63.58 ± 6.95%; and 63.08 ± 1.92% (Nunes et al., 2016, 2019; Almeida-Monteiro et al., 2017). These values agree with what was found in the present study for the control group (64.80 ± 5.86%). However, it is possible to notice higher values for treatments containing egg yolk (between 84% and 91%), close to the result for fresh sperm (96.70 ± 0.45%). In the present research, treatments containing egg yolk resulted in higher membrane integrity percentages than all the other treatments, while the treatments containing lecithin had the lowest ones. In addition, egg yolk treatments at any concentration, provided more protection to the plasma membrane than the control group, which shows great advantage brought by its use.

This result differs from what was found when cryopreserving *C. macropomum* sperm with 2.5% soy lecithin, as this concentration resulted in higher membrane integrity rates than egg yolk and control (Lopes, 2019). Furthermore, when using 5%, 10% and 15% soy lecithin and comparing them with egg yolk, Yildiz et al. (2013) found no statistical difference for this parameter. This shows, once again, the species-specific characteristic of cryoprotective substances.

The many benefits of egg yolk are mainly attributed to its composition rich in LDL, which makes it capable of interacting with plasma membrane lipids. In studies with mammals, it is estimated that yolk LDL may act by releasing phospholipids which will replace phospholipids lost by the sperm membrane (Moussa et al., 2002). However, when using LDL extracted from egg yolk in *S. cettii* sperm cryopreservation and comparing it with whole egg yolk, Rusco et al. (2019) observed that the isolated lipoproteins had lower results for several parameters, including membrane integrity, DNA integrity and motility rate and duration.

Furthermore, it is assumed that the lipid portion of the lipid-protein complex extracted from egg yolk is what makes it capable of protecting sperm against cold shock and, thus, acting as a resistance factor (Kampschmidt et al., 1953). This portion is made up of phospholipids and lecithin. On the other hand, additional substances present in the egg yolk may act to preserve the sperm during storage at low temperatures (Kampschmidt et al., 1953). This indicates that yolk has other components that act positively in protecting the cell. Among them, we can highlight substances with antioxidant potential such as free aromatic amino acids, which can bestow egg yolk a role against oxidative damage (Nimalaratne et al., 2011).

## Conclusion

The results obtained in this research indicate that, among the compounds tested, when compared to control, egg yolk did not bring unwanted effects to important variables observed for assessing sperm quality. Additionally, egg yolk treatments improved protection to plasma membrane. Among treatments with egg yolk (5%, 10% and 12%), there were no statistical significance between them, therefore, 5% concentration would suffice to observe desirable attributes on sperm quality.

Hence, we conclude that 5% egg yolk associated with 10% Me_2_SO and 5% Glucose is the most suitable solution for the *P. brevis* sperm cryopreservation.
